# Primed for cancer: Li Fraumeni Syndrome and the pre-cancerous niche

**DOI:** 10.3332/ecancer.2015.541

**Published:** 2015-05-21

**Authors:** Pan Pantziarka

**Affiliations:** The George Pantziarka TP53 Trust, London KT1 2JP, UK; Anticancer Fund, Brussels, 1853 Strombeek-Bever, Belgium

**Keywords:** TP53, Li Fraumeni Syndrome, cancer predisposition, pre-cancerous niche

## Abstract

The complex relationship between tumour and stroma is still being elucidated but it is clear that cancer is a disease of more than just malignant cells. However, the dominant focus of our current understanding of Li Fraumeni Syndrome (LFS) remains on the function of p53 as ‘guardian of the genome’. Recent evidence shows that the TP53 gene is at the nexus of a wider range of functions, including aspects of cellular metabolism, aging and immunity. Incorporating this broader picture of the role of TP53 together with our understanding of the role of the host microenvironment in cancer initiation and progression gives a more nuanced picture of LFS. Furthermore, there is clinical evidence to suggest that the host environment in healthy individuals with LFS already includes some of the features of a ‘pre-cancerous niche’ that makes cancer initiation more likely. It is suggested, finally, that there are pharmacological interventions capable of altering this pre-cancerous niche, thus potentially reducing the cancer risk in individuals with LFS.

## Introduction

Li Fraumeni Syndrome (LFS) is a rare autosomally dominant genetic syndrome associated with a highly elevated risk of developing cancer. First identified in a group of families with an unusually increased pattern of cancer incidence by Frederick Li and Joseph Fraumeni in 1969, the syndrome is characterised by early development of breast cancer and a range of sarcomas, adrenocortical carcinoma, gliomas, leukaemias, and other cancers, particularly in children and early adulthood [1, 2]. LFS is associated with germ-line TP53 tumour suppressor gene mutations, although it is estimated that around 20–30% of individuals diagnosed with the condition do not carry such a mutation [3, 4].

Breast cancer, very often pre-menopausal, is the most common tumour in women with LFS, with an estimated risk of 49% by the age of 60, with one recent study finding the median age of diagnosis as 32 years (range 22–46) [5]. Around 50% of sufferers will develop cancer before the age of 30 [6], while the life-time risk of developing cancer has been estimated at 70% for males and 100% for women [7]. The most common cancers are breast cancer (27%) and soft-tissue and bone sarcomas (25%) [6].

A number of different diagnostic criteria exist for LFS, and in addition to ‘classical LFS’, there is a recognised Li Fraumeni-like (LFL) Syndrome, which has less stringent diagnostic and testing criteria. The different criteria are discussed in [6] and [8]. The management of LFS post-diagnosis is broadly consistent globally. There is an emphasis on active surveillance, with new protocols being trialled in different parts of the world (for example NCT01464086 and NCT01737255), and women are offered the opportunity to have risk-reducing double mastectomy. Many of the new surveillance protocols under investigation focus on the use of regular whole-body MRI, ultrasound and other clinical investigations that do not impose additional exposure to ionising radiation. Results from some studies of such protocols have shown that active surveillance can lead to significant survival benefits, for example one small non-randomised cohort study found that patients in the active surveillance group had a 100% three-year survival, in contrast to a 21% survival in a matched non-surveillance group [9]. Aside from the option of mastectomy for women, there are no other active cancer-prophylactic measures in clinical use or currently being trialled.

Whilst TP53 is the gene most associated with LFS, there is evidence that some individuals harbour mutations in related genes. For example analysis of non-TP53 mutant LFS families showed a greater incidence of a single-nucleotide polymorphism 309 in the MDM2 gene than in the general population [10]. MDM2 is a key negative regulator of TP53, and this particular polymorphism results in increased expression of MDM2 and a consequent attenuation of the p53 pathway. While there is animal data suggesting that MDM2 polymorphisms may be associated with elevated cancer incidence [11], the prognostic significance of mutations in other genes in the TP53 pathway have yet to be established in humans.

LFS is not the only disease associated with TP53 abnormalities. There is increasing evidence of a role of defective p53 activity in the pathophysiology of a number of other rare genetic conditions, including Diamond-Blackfan anemia (DBA), CHARGE syndrome and ATR-Seckel syndrome. In these diverse syndromes, which are associated with a range of congenital abnormalities, altered p53 activity is implicated in certain stem cell niches [12–14].

Because of the centrality of the p53-associated tumour suppressor pathway, carcinogenesis in LFS is viewed primarily through the lens of genetic damage. It is assumed that in LFS cancer develops because of additional genetic damage to the TP53 gene causing loss of heterozygosity (LOH) and/or gain of function (GOF), leading to malignant transformation [15–17]. In effect cancer incidence in LFS is due to the ‘two-hit’ model first proposed by Knudson based on analysis of hereditary transmission of retinoblastoma [18]. One immediate consequence of this model is a caution in treating LFS patients with radiotherapy or limiting exposure to ionising radiation during diagnostic investigation [6, 19].

In recent years our understanding of the role of p53 has expanded considerably, and now includes key functions in aging [20, 21], cellular metabolism [22, 23], regulation of homeostasis [24] and immune function [25, 26]. Research has also focused on the non-cell-autonomous functions of p53 which can suppress tumourigenesis by the promotion of an anti-tumour microenvironment [27, 28]. By the same token, just as the apoptotic program can be subverted, so too can the non-cell-autonomous functions be subverted to promote a pro-tumourigenic microenvironment. This complex interplay between p53 and the microenvironment is central to the thesis outlined in this paper.

We are also beginning to explore the complex landscape of TP53 mutations and the role this plays in cancer initiation, progression and treatment response [29–31]. Similarly the study of the impact of p53 isoforms is at an early stage of development, but it is already clear that different isoforms can radically alter transcriptional activity and core functionality, again differentially impacting cancer initiation, progression and response to treatment [32, 33].

In the same period we have also seen an increased understanding of the role of the microenvironment in cancer initiation, progression and metastasis [34–36]. Where once cancer was seen as primarily a disease of mutated cells, it is now increasingly being viewed as a dynamic evolutionary system that incorporates stromal cells, immune infiltrates, tumour vasculature and heterogenic clonal subpopulations of cancer cells [37, 38].

These new insights into the functionality of p53 and the broader understanding of tumour biology have yet to be integrated into our understanding of cancer initiation in individuals with LFS. This paper will attempt to relate this new knowledge with a number of clinical features common in individuals with LFS and to shed light on the implications these might have on cancer risk. In particular it will propose a more nuanced view of carcinogenesis in LFS, suggesting that there are important additional factors over and above genetic ablation of p53 tumour suppressor function.

## The importance of the host environment

Stephen Paget, building on work by Ernst Fuchs, proposed the ‘seed and soil’ hypothesis in 1889 to explain the tendency of breast cancers to metastasise preferentially to certain tissues [39]. He suggested that the ‘soil’ (host environment) was as important as the ‘seed’ (the migrating tumour cell) in the complex process of metastasis, and that certain tissues (bone, brain, liver) provide a more hospitable environment for cancer cells to take ‘root’ and produce viable tumours. While the hypothesis directs attention to the network of relationships between migrating tumour cells and the various elements of the host environment, it is also a reminder that there is much still to be explained about the process of metastatic spread of disease. Many key controversies remain to be resolved, for example do cancer cells acquire metastatic potential early or late in the process of tumourigenesis? Where initial theory suggested that metastatic potential was acquired at a late stage in primary cancer growth, driven by the accumulation of genetic changes and clonal evolution, newer theories suggest that it is in fact a property of certain subtypes of cancer cells and that these cells have an intrinsic ability to disseminate to and proliferate in distant sites [40]. Of more consequence to the thesis outlined in this paper, there has been a greater interest in understanding the nature of the ‘soil’, in particular in understanding what it is that makes certain tissues more conducive to tumour growth than others.

An important stream of work has explored the idea of a ‘pre-metastatic niche’, an idea first elucidated by David Lyden and colleagues in 2005–2006 [41, 42]. In this model it is not the intrinsic properties of the migrating tumour cell that determines the success or otherwise of the metastatic process, but the properties of the host microenvironment in which the tumour cell lodges. A permissive environment will provide the growth factors, nutrient flow and support systems that enable the tumour cell to proliferate and grow into a viable metastatic nodule. In contrast, an inhospitable host environment will not provide the factors that the tumour cell needs to survive and proliferate, and therefore no metastatic growth will form regardless of the properties of the tumour cell. Furthermore, it appears that there are certain tissues and microenvironments which are more likely to harbour these pre-metastatic niches, and that these tissues correspond to the most likely sites of metastatic spread for different cancer types.

Analysis of the process of metastasis shows that certain cell types, in particular hematopoietic progenitor cells (HPC) expressing VEGFR-1 become clustered at sites *prior* to the arrival of tumour cells, a finding confirmed in breast cancer patients in addition to murine models [41]. The clustering of these VEGFR-1+ HPCs initiates a cascade that includes the expression of integrins (including VLA-4), matrix metalloproteinase 9 (MMP9), and downstream expression of VEGF-A and fibronectin expression in resident fibroblasts. Furthermore, in addition to local tissue remodelling, there is evidence that these clusters promote the chemoattraction and attachment of circulating tumour cells and endothelial progenitor cells (EPC), which are essential for angiogenesis to take place [43].

Formation of these clusters of VEGFR-1+ HPCs is triggered by the release of factors from the primary tumour with homing to specific sites related to ligand-receptor pairing. For example breast cancer cells expressing the chemokine receptor CXCR4 home to organs with high levels of its ligand SDF-1 (stromal derived factor-1), for example the bone marrow, lungs and liver [43]. In a murine model it has been shown that the inflammatory chemoattractants S100A8 and S100A9 released from primary B16 melanoma tumours pre-condition the lungs, create pre-metastatic niches and attract both tumour and inflammatory cells [44].

McAllister and colleagues investigated mechanisms by which primary tumours in a mouse model are able to ‘condition’ distant tissues to create conditions in which otherwise indolent tumour cells became activated through the recruitment of a reactive stroma, a process they termed systemic instigation [45, 46]. However, both the primary tumour and the indolent tumour cells were implanted in the animal model used, and therefore this conditioning of a metastatic niche was co-incident with tumour cell inoculation. Similarly Massagué *et al* identified cancer-cell expression of tenascin-C (TNC), an extracellular matrix (ECM) protein of stem cell niches, as essential for the establishment of breast cancer metastases in the lungs [47]. Initial expression of TNC by breast cancer cells was required to maintain viability in the lung parenchyma, through up-regulation of Wnt and Notch signalling, until stromal expression of TNC by infiltrating myofibroblasts or other stromal sources accumulated in the expanding nodules to provide a supportive metastatic stem cell niche.

Liu and colleagues used a mouse of breast cancer metastasis to the lungs to show that primary tumours were able to induce inflammatory changes via release of VEGF and consequent recruitment of bone-marrow-derived cells in the lungs [48]. Furthermore VEGF induced prostaglandin E2 (PGE2) production in pulmonary endothelial cells and enhanced the adhesion of injected circulating tumour cells. Analysis showed that the sites of inflammatory response in the lungs preferentially harboured injected tumour cells, showing that the primary tumour was able to induce pre-metastatic niche formation.

A recent review by McAllister and Weinberg summarises additional evidence showing that primary tumours can condition distant tissues, including discussion of pre-metastatic niche creation [49].

A key feature of this pre-metastatic niche is the inclusion of multiple stromal cell types, including fibroblasts, macrophages and other immune cells, HPCs and EPCs. These are, of course, also many of the components of the *primary* tumour microenvironment (TME) [50]. Increasingly our view of cancer is that of an evolving and dynamic ecosystem that incorporates tumour and non-tumour cells in complex patterns of competition and cooperation. No longer focused purely on the genomic changes within the tumour cell, viewing cancer as an ecosystem redirects attention to those factors in the microenvironment which support survival, proliferation and ultimately the metastatic process [37, 51, 52].

Significantly, an understanding of the importance of the TME to disease progression, resistance to treatment and metastatic dissemination also creates new opportunities for therapeutic intervention [53]. Altering aspects of the TME, by targeting specific populations of stromal cells or signalling pathways, can interfere with the ecological balance within the tumour and render cancer more immunogenic, less resistant to chemotherapy or radiation and so on.

## The pre-cancerous niche

An extension of the idea of the pre-metastatic niche to primary disease has also been discussed [54]. A multi-step model of primary carcinogenesis has been proposed, starting with niche construction leading to expansion and maturation. According to this model, tumourigenesis cannot take place without the existence of the pre-cancerous niche, regardless of the accumulated genetic mutations in a transformed cell. Numerous experiments have indeed shown that malignant cells transplanted into non-transformed tissues (in other words in tissues lacking the pre-cancerous niche) experience loss of malignant behaviour [55–57]. Initiation of niche construction may have multiple physiological causes, including the local action of carcinogenic agents, tissue injury, infection or aging [54].

A common feature of the pre-cancerous niche, independent of the initial cause, is the presence of chronic inflammation. The link between cancer and inflammation is well-characterised, and it is being increasingly recognised as causative rather than a by-product of disease progression [58–60]. Chronic inflammation is associated with cancer incidence related to bacterial and viral infections, chronic disease (liver cirrhosis, diabetes), tobacco-smoke inhalation, obesity and aging. It is also associated with the expansion and maturation of the pre-cancerous niche, with the recruitment of inflammatory cells, activation and remodelling of stromal components (particularly fibroblasts), release of pro-angiogenic factors and the homing of transformed cells [35, 36]. Tumour formation occurs through the co-evolution of the niche and homed transformed cells, creating a tumour microenvironment that supports further proliferation and invasion of the complex and co-evolving mass.

It is clear that this complex process, which has yet to be fully elucidated, incorporates many of the hallmarks of cancer [61]. It is also clear that it is a challenge for researchers in LFS to incorporate this complex picture into our understanding of cancer initiation in people with this condition.

## *TP53* and the pre-cancerous niche

It is our contention that people with LFS are ‘primed’ for cancer initiation because a number of the key drivers of niche initiation are a feature of the *non-cancerous* LFS host. That is, they are at greater risk of cancer initiation because of more than defective tumour suppressor activity. In other words many of the other functions of the p53 network are also important drivers of cancer risk. Equally important, it is possible that these other functions, many of which directly impact the formation of the pre-cancerous niche, are amenable to drug targeting in a way that the pro-apoptotic function of p53 signalling is not.

**Figure 1.**
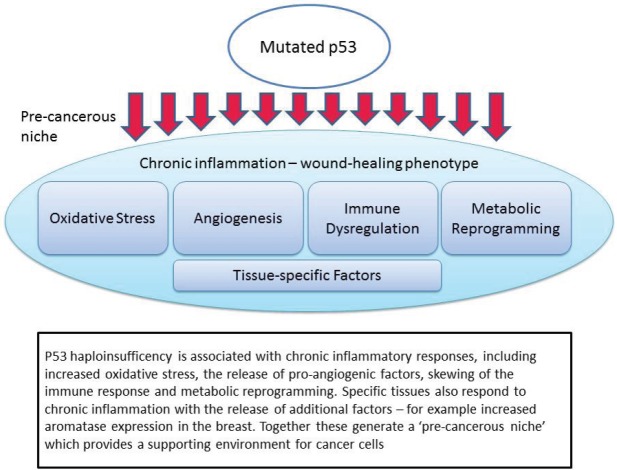
Mutated p53 function leads to the creation of a pre-cancerous niche.

The non-apoptotic functions which are important in this context are: chronic inflammation and oxidative stress; pro-angiogenic signalling; immune dysregulation; metabolic reprogramming; and tissue-specific interactions (Figure 1). Each of these will be discussed in turn, noting both the evidence from recent studies in p53, but also relevant clinical evidence from LFS patients or appropriate animal models.

### Chronic inflammation and oxidative stress

The role of chronic inflammation in cancer initiation and progression is well-known and well-characterised [46]. Inflammatory cells, including neutrophils, monocytes, macrophages, mast cells and lymphocytes, are recruited to the site of the inflammatory response. Histamine is released causing increased vasodilation. Pro-angiogenic factors are released and extensive remodelling of the tissues takes place. Reactive oxygen species (ROS), produced by multiple inflammatory cell types, further drive inflammation in a positive feedback loop [59]. The p53 network is activated during this inflammatory state, most likely in direct response to the cellular stresses invoked by elevated ROS levels [63, 64].

However, there is also evidence that loss of p53 function can itself act as a *driver* of inflammation in an NF-kB-dependent manner [65]. Significantly, this study showed that loss of p53 alone was insufficient to cause tumourigenesis. The relationship between increased inflammation, oxidative stress and p53 is apparent in numerous pre-cancerous or inflammatory conditions [66–69]. Furthermore, there is also evidence that cancer-free LFS sufferers exhibit clinical signs of increased levels of oxidative stress compared to a paired group of family members without TP53 mutations [70].

### Angiogenesis

Tumour neo-angiogenesis is an important rate-limiting step in tumour growth and progression. In terms of the pre-cancerous niche and LFS, there are two elements that are of interest in the development of a pro-angiogenic environment. In the first case the increased oxidative stress associated with chronic inflammation acts to induce angiogenesis as part of a ‘wound healing’ response [71, 72]. However, p53 is also known to have an influence on angiogenesis via thrombospondin-1 (TSP-1), which acts as an endogenous anti-angiogenic factor [73, 74]. Loss of p53 leads to down-regulation of TSP-1 which correlates to increased expression of VEGF and other pro-angiogenic signals [75, 76].

There is also some evidence that a number of micro-RNAs, some of which are involved in the tumour suppressive and stress response functions of p53, have an influence on angiogenesis [77]. For example miR-107 mediates the p53 response to hypoxia by suppressing the expression of hypoxia inducible factor-1β (HIF-1β), which in turn down-regulates expression of VEGF [78]. Impaired transcriptional activity in mutant p53 may therefore lead to loss of anti-angiogenic activity and increased expression of VEGF and angiogenesis [79].

The evidence from fibroblasts derived from LFS patients confirms that loss of the wild-type p53 allele is sufficient to decrease TSP-1 expression and an increase in VEGF [73]. A step-wise process may take place whereby additional changes, such as oncogene activation, may lead to a more fully pro-angiogenic phenotype [80]. Additionally it should be noted that there is evidence that some gain-of-function (GOF) TP53 mutations (including R175H and R273H common in people with LFS) have been shown to have tumour angiogenesis promoting activity [81, 82].

### Immune Dysregulation

Little work has been done to investigate the effect that lack of wild-type p53 in stromal cells has on immune response to tumour growth. One notable exception compared the rate of tumour development in mice with different p53 status, observing that implanted B16F0 melanoma tumours grew at a faster rate in mice lacking wild-type p53 [83]. Furthermore, the effect was not apparent in severe combined immunodeficiency mice, suggesting that this difference in tumour growth is immune-related. Tumours were also implanted in wild-type mice along with mesenchymal stem cells (MSC), a major part of the microenvironment, with differing p53 status. Wild-type mice co-implanted with stromal cells lacking p53 developed larger tumours than mice with wild-type cells in the stroma. The p53-deficient MSC cells inhibited T-cell function, over-expressed inducible Nitric Oxide synthase and generated an immunosuppressive microenvironment conducive to tumour growth.

There is also an influence on immune response via p53 regulation of toll-like receptor (TLR) expression [84]. TLRs, the pattern recognition receptors involved in the innate immune response, are able to respond to both exogenous and endogenous ligands, are also now recognised to have a role in adaptive immunity [85]. Furthermore, TLRs, which are now known to be expressed by cancer cells in addition to a range of non-cancer cells [86], have a complex role in cancer, with both pro- and anti-cancer activity [87, 88]. There is some evidence that TLR signalling plays an active role in carcinogenesis during chronic inflammation, particularly with respect to facilitating escape from immune surveillance, release of pro-inflammatory cytokines and chemokines and tumour angiogenesis [86, 89]. TLR expression is modulated by p53 activation, and there is evidence that TP53 mutants, including a number associated with germ-line mutations, can differentially impact TLR expression in response to cellular stressors [84, 90].

There is also emerging evidence that p53 has a role in the regulation of PDL1 (programmed death ligand 1), a transmembrane protein expressed by tumour cells that is the target of intense scientific interest in relation to its role in disarming anti-tumour immune responses in a range of malignancies [91]. Data presented by Cortez and colleagues at AACR 2015 showed that PDL1 is regulated by p53 via miR-34a in NSCLC [92].

### Metabolic Reprogramming

**Figure 2.**
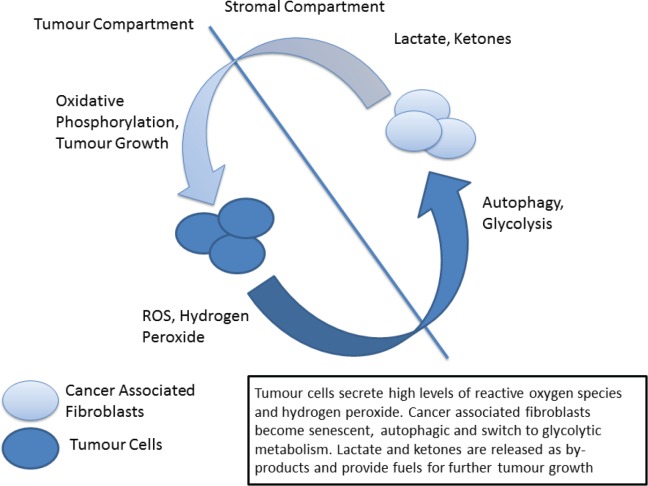
The ‘reverse Warburg’ phenotype.

Metabolic reprogramming is another of the hallmarks of cancer in which p53 plays a central role [93, 94]. The traditional view, termed the Warburg Effect, focuses on the increased metabolic needs of malignant tissues and a consequent metabolic switch to glycolysis in tumours. The Warburg effect manifests itself as an increase in glucose metabolism by tumour cells, the generation of lactate as a by-product and an increase in acidity and hypoxia, both driving clonal evolution towards a more malignant phenotype. However, in recent years this view has been challenged by the emergence of the ‘reverse Warburg effect’ hypothesis in which distinct metabolic compartments exist within the tumour and stromal cell populations [95, 96]. In this model increased oxidative stress drives cancer associated fibroblasts to switch to glycolytic metabolism, producing lactate and other by-products which are metabolised by tumour cells – effectively creating a metabolic shuttle between tumour and stroma (Figure 2) [97]. Analysis of patient samples in a variety of cancer types has indicated the presence of both ‘Warburg’ and ‘reverse Warburg’ phenotypes, suggesting that cancer cells are metabolically plastic and can adapt and change during disease progression [98, 99]. Of note the ‘reverse Warburg’ phenotype has also been detected in osteosarcoma, one of the ‘signature’ cancers associated with LFS [100].

It has previously been proposed that cancer initiation in LFS is related to this ‘two compartment model’ of tumour metabolism [101]. Briefly, p53 signalling in response to increased oxidative stress can trigger cellular autophagy in fibroblastic cells, shifting their metabolism towards glycolysis. The increase in secretion of lactate drives further changes in the microenvironment, producing an immunosuppressive and pro-tumour environment. Cells may also react to environmental and metabolic stresses by undergoing senescence, again mediated by p53, eventually becoming immortalised and/or undergoing malignant transformation. When these cells become transformed it is in a hospitable microenvironment that has been ‘primed’ for cancer initiation.

A key marker for the ‘reverse Warburg’ or ‘two-compartment’ phenotype is loss of stromal cav-1 expression [102], and this finding has been confirmed in people with LFS compared to non-affected family members [103].

### Tissue-specific factors

While the factors listed above are common to pre-cancerous niches in multiple tissue types, there are also factors which are specific to some tissues and not others. This accords with the observations both in primary disease and in the pattern of metastatic spread preferentially to given tissues, for example bones, lungs and liver in breast cancer. In particular cancer incidence in LFS is more strongly associated with certain tissues (e.g. breast, sarcomas, adrenocortical carcinoma) than with others (e.g. lung, bladder). It is hypothesised that this pattern of primary tumour incidence is related to secreted factors in specific tissues which contribute to pre-cancerous niche formation.

An analysis of the breast cancer phenotype in women with LFS found that 84% of invasive tumours were hormone responsive (ER and/or PR), with a majority of these also being positive for Her2/neu, figures which are higher than for a comparable non-LFS population [5]. Evidence that this may be related to factors in stromal tissues comes from a recent analysis of the relationship between stromal aromatase expression, which is associated with ER+ breast cancer, and p53 in breast tissue [104]. The authors analysed breast adipose tissue to examine the relationship between aromatase expression, prostaglandin E2 (PGE2) and p53. Results indicated a feedback loop between p53 and the expression of aromatase and that low p53 expression caused increased aromatase. This relationship between aromatase and p53 was shown to be mediated by PGE2, which down-regulates p53 expression. Significantly, analysis of tissue samples from women with LFS showed statistically significantly higher levels of aromatase in tumour-associated stromal adipose tissue compared to non-LFS women.

## A different view of cancer initiation

The pre-cancerous niche is, as we have seen, a hospitable and conducive environment in which tumours can take root and prosper. A germ-line TP53 mutation leads to many of the key features of such a niche being expressed in otherwise healthy and non-cancerous LFS individuals. Many of the features of this niche environment are self-reinforcing or coupled in positive feedback loops such that inflammatory and pro-tumour signalling is perpetuated leading to chronic inflammation.

The question that arises is whether this pre-cancerous niche is itself a driver of carcinogenesis in people with LFS. Again, the lack of functioning wild-type p53 may conspire to initiate transformation and carcinogenesis. It is known that telomere length is shorter in people with LFS compared to non-affected family members [105, 106], and that this may be related to the age of cancer onset [107, 108]. Progressive shortening of telomeres, a process that is exacerbated by oxidative and other cellular stresses, eventually leads to ‘telemore crisis’ and consequent DNA damage. At this stage the p53 damage response may lead to senescence or apoptosis, and indeed there is evidence that cells from LFS patients display greater levels of DNA damage (chromosomal instability, senescence etc) [109–111].

**Figure 3.**
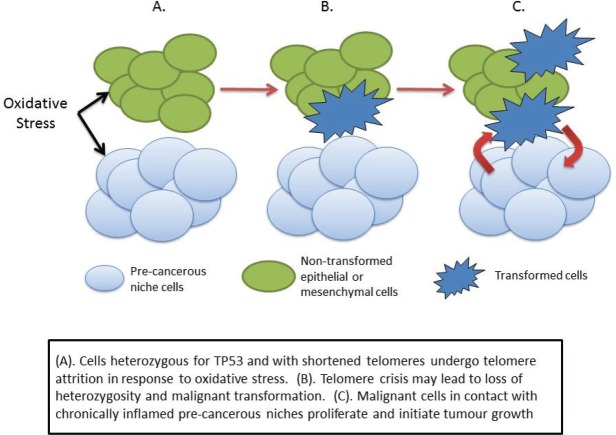
Cancer initiation in LFS as a consequence of haploinsufficiency of p53.

We may speculate, therefore, that the combination of lack of wild-type p53 and increased oxidative stress may therefore cause the subsequent genetic damage leading to cellular transformation and cancer initiation. One possible scenario is that the increased oxidative stress in the pre-cancerous niche causes telomere shortening in adjacent non-stromal cells, leading to crisis and initiating a sequence that eventually leads to malignant transformation (Figure 3). Transformed cells in contact with the pre-cancerous niche find a hospitable ‘soil’ in which they ‘seed’ tumour growth.

This differs from the conventional view of carcinogenesis in LFS being caused by a *random* ‘second hit’ mutation leading to cancer. Instead it suggests very strongly that the phenotypic features of the LFS host which are caused by the germ-line mutation in TP53 creates a set of pre-cancerous niches which may act to *cause* the ‘second hit’ or the switching on of oncogenic pathways.

Supporting evidence for this hypothesis comes from a series of animal experiments in a mouse model of LFS [112]. Heterozygous and wild-type *Trp53* mice were treated with either surgical implantation of a foreign object to induce chronic inflammation, or a sham operation. Thirty of 38 (79%) heterozygous mice developed sarcomas around the implant at a mean age of 46 weeks, compared to one (10%) of the wild-type mice at 56 weeks. No sarcomas developed at the sites of sham operation. Two of 10 (20%) control heterozygous mice (no implant) also developed sarcomas, but at a mean age of 80 weeks. In 90% of implant-induced sarcomas, loss of heterozygosity was observed suggesting a causative effect from the chronic inflammation induced by the implant. This is in contrast to the work of Lyden and colleagues in that this was a model of *primary* carcinogenesis rather than metastatic spread through the creation of a pre-metastatic niche by the primary tumour.

Of note it is the *phenotypic* features of the host which are important drivers of this process. These features may also be present in people diagnosed with LFS or LFS-like conditions in which there is no TP53 mutation detected. It is possible that a similar process takes place in other cancer predisposition syndromes in addition to LFS. There may be a number of different genetic drivers which can lead to the creation of pre-cancerous niches in Cowden Syndrome (associated with the PTEN tumour suppressor), Peutz–Jeghers Syndrome (associated with STK11/LKB1 gene) and other such conditions. Further investigation of this hypothesis is warranted as it may mean that similar clinical strategies may apply to a range of syndromes normally viewed and treated in isolation.

## Conclusion

In widening our view of cancer initiation in LFS to include the non-apoptotic functions of the TP53 gene we can see that the microenvironment assumes a much greater significance. This has important clinical implications for people with LFS for it is known that some of the features of the micro-environment may be more easily amenable to drug targeting than p53 itself. Indeed some pre-clinical work in animal models has already produced intriguing results.

For example, focusing purely on metabolic plasticity, Komarova and colleagues showed that the mTOR inhibitor rapamycin, which can act to inhibit senescence, increased the life-span and delayed tumourigenesis in mice bearing heterozygous p53 mutations [113]. In terms of the ‘two compartment’ model of tumour metabolism, inhibiting senescence in stromal cells may lead to fewer cells switching to glycolysis and thus depriving coupled tumour cells of lactate and other high-energy fuels. Similarly, caloric restriction in a mouse model of LFS also delayed tumour onset in adult mice [114], which may be related to reduced supply of glucose feeding glycolytic cells.

However, there are other features of the pre-cancerous niche which can also be targeted. Reductions in chronic inflammation or oxidative stress may also be possible. For example the anti-diabetic drug metformin has pleiomorphic effects which may be suitably exploited to attack the pre-cancerous niche in multiple ways:

- Reduction in oxidative stress
- Reduction in chronic inflammation
- Action on metabolic pathways in tumour and/or stromal cells
- Reduction of hepatic glucose production
- Pro-apoptotic effects in some cancers
- Increase in anti-tumour immunity [115]

A range of other drugs, many of which are being actively investigated as possible repurposed anti-cancer drugs [116] also show some promise, including aspirin and other NSAIDs and the lipophilic statins. Other strategies may include dietary and other interventions to reduce oxidative stress, chronic inflammation or increased telomere attrition.

Investigation of the cancer-preventative effect of these drugs in relevant animal models is warranted, as is further investigation of the features of the pre-cancerous niche in people with LFS. It may be that there is prognostic significance in measures of oxidative stress, level of cav-1 expression or other biomarkers related to chronic inflammation.

Clinical trials of cancer prevention in LFS are problematic given the relatively large sample sizes required to show efficacy of any interventions. However, if the pre-cancerous niche hypothesis proves to be equally applicable to other cancer predisposition syndromes then an increased pool of patients may mean that clinical trials may become more feasible. It is suggested therefore, that further investigation of this hypothesis be undertaken in other cancer predisposition syndromes.

## Competing interests

The author declares that there are no competing interests. This research was funded by the George Pantziarka TP53 Trust.

## References

[1] Li FP, Fraumeni JF (1969). Soft-tissue sarcomas, breast cancer, and other neoplasms A familial syndrome?. Ann Intern Med.

[2] Malkin D (2011). Li-Fraumeni syndrome. Genes Cancer.

[3] Malkin D, Li FP, Strong LC (1990). Germ line p53 mutations in a familial syndrome of breast cancer, sarcomas, and other neoplasms. Science.

[4] Nichols KE, Malkin D, Garber JE (2001). Germ-line p53 mutations predispose to a wide spectrum of early-onset cancers. Cancer Epidemiol Biomarkers Prev.

[5] Masciari S, Dillon DA, Rath M (2012). Breast cancer phenotype in women with TP53 germline mutations: a Li-Fraumeni syndrome consortium effort. Breast Cancer Res Treat.

[6] McBride KA, Ballinger ML, Killick E (2014). Li-Fraumeni syndrome: cancer risk assessment and clinical management. Nat Rev Clin Oncol.

[7] Chompret A, Brugières L, Ronsin M (2000). P53 germline mutations in childhood cancers and cancer risk for carrier individuals. Br J Cancer.

[8] Kamihara J, Rana HQ, Garber JE (2014). Germline TP53 mutations and the changing landscape of Li-Fraumeni syndrome. Hum Mutat.

[9] Villani A, Tabori U, Schiffman J (2011). Biochemical and imaging surveillance in germline TP53 mutation carriers with Li-Fraumeni syndrome: a prospective observational study. Lancet Oncol.

[10] Ruijs MWG, Schmidt MK, Nevanlinna H (2007). The single-nucleotide polymorphism 309 in the MDM2 gene contributes to the Li-Fraumeni syndrome and related phenotypes. Eur J Hum Genet.

[11] Post SM, Quintás-Cardama A, Pant V (2010). A high-frequency regulatory polymorphism in the p53 pathway accelerates tumor development. Cancer Cell.

[12] Dutt S, Narla A, Lin K (2011). Haploinsufficiency for ribosomal protein genes causes selective activation of p53 in human erythroid progenitor cells. Blood.

[13] Van Nostrand JL, Brady CA, Jung H (2014). Inappropriate p53 activation during development induces features of CHARGE syndrome. Nature.

[14] Lee Y, Shull ER, Frappart PO (2012). ATR maintains select progenitors during nervous system development. EMBO J.

[15] Srivastava S, Zou ZQ, Pirollo K (1990). Germ-line transmission of a mutated p53 gene in a cancer-prone family with Li-Fraumeni syndrome. Nature.

[16] Rivlin N, Brosh R, Oren M (2011). Mutations in the p53 tumor suppressor gene: important milestones at the various steps of tumorigenesis. Genes Cancer.

[17] Parant JM, George SA, Holden JA (2010). Genetic modeling of Li-Fraumeni syndrome in zebrafish. Dis Models Mech.

[18] Knudson AG (1971). Mutation and cancer: statistical study of retinoblastoma. Proc Natl Acad Sci USA.

[19] Heymann S, Delaloge S, Rahal A (2010). Radio-induced malignancies after breast cancer postoperative radiotherapy in patients with Li-Fraumeni syndrome. Radiat Oncol.

[20] Rufini A, Tucci P, Celardo I (2013). Senescence and aging: the critical roles of p53. Oncogene.

[21] Sahin E, De Pinho RA (2012). Axis of ageing: telomeres, p53 and mitochondria. Nat Rev MolCell Biol.

[22] Wang SJ, Gu W (2014). To be, or not to be: functional dilemma of p53 metabolic regulation. Curr Opin Oncol.

[23] Cheung EC, Vousden KH (2010). The role of p53 in glucose metabolism. Curr Opin Cell Biol.

[24] Zhuang J, Ma W, Lago CU (2012). Metabolic regulation of oxygen and redox homeostasis by p53: lessons from evolutionary biology?. Free Radical Bio Med.

[25] Menendez D, Shatz M, Resnick MA (2013). Interactions between the tumor suppressor p53 and immune responses. Current Opin Oncol.

[26] Iannello A, Thompson TW, Ardolino M (2013). p53-dependent chemokine production by senescent tumor cells supports NKG2D-dependent tumor elimination by natural killer cells. J Exp Med.

[27] Lujambio A, Akkari L, Simon J (2013). Non-cell-autonomous tumor suppression by p53. Cell.

[28] Bieging KT, Mello SS, Attardi LD (2014). Unravelling mechanisms of p53-mediated tumour suppression. Nat Rev Cancer.

[29] Monti P, Perfumo C, Bisio A (2011). Dominant-negative features of mutant TP53 in germline carriers have limited impact on cancer outcomes. Mol Cancer Res.

[30] Garritano S, Inga A, Gemignani F (2013). More targets, more pathways and more clues for mutant p53. Oncogenesis.

[31] Bisio A, Ciribilli Y, Fronza G (2014). TP53 mutants in the tower of babel of cancer progression. Hum Mutat.

[32] Surget S, Khoury MP, Bourdon JC (2013). Uncovering the role of p53 splice variants in human malignancy: a clinical perspective. Onco TargetsTher.

[33] Senturk S, Yao Z, Camiolo M (2014). P53Ψ Is a transcriptionally inactive P53 isoform able to reprogram cells toward a metastatic-like state. Proc Natl Acad Sci USA.

[34] Allen M, Jones LJ (2011). Jekyll and Hyde: the role of the microenvironment on the progression of cancer. J Pathol.

[35] Quail DF, Joyce JA (2013). Microenvironmental regulation of tumor progression and metastasis. Nat Med.

[36] Hanahan D, Coussens LM (2012). Accessories to the Crime: functions of cells recruited to the tumor microenvironment. Cancer Cell.

[37] Pienta KJ, McGregor N, Axelrod R (2008). Ecological therapy for cancer: defining tumors using an ecosystem paradigm suggests new opportunities for novel cancer treatments. Transl Oncol.

[38] Tian T, Olson S, Whitacre JM (2011). The origins of cancer robustness and evolvability. Integr Biol.

[39] Paget S (1889). The distribution of secondary growths in cancer of the breast. Lancet.

[40] Coghlin C, Murray GI (2010). Current and emerging concepts in tumour metastasis. J Pathol.

[41] Kaplan RN, Riba RD, Zacharoulis S (2005). VEGFR1-positive haematopoietic bone marrow progenitors initiate the pre-metastatic niche. Nature.

[42] Kaplan RN, Rafii S, Lyden D (2006). Preparing the ‘soil’: the premetastatic niche. Cancer Res.

[43] Psaila B, Kaplan RN, Port ER (2007). Priming the ‘soil’ for breast cancer metastasis: the pre-metastatic niche. Breast Dis.

[44] Hiratsuka S, Watanabe A, Aburatani H (2006). Tumour-mediated upregulation of chemoattractants and recruitment of myeloid cells predetermines lung metastasis. Nat Cell Biol.

[45] Elkabets M, Gifford AM, Scheel C (2011). Human tumors instigate granulin-expressing hematopoietic cells that promote malignancy by activating stromal fibroblasts in mice. J Clin Invest.

[46] Kuznetsov HS, Marsh T, Markens BA (2012). Identification of luminal breast cancers that establish a tumor-supportive macroenvironment defined by proangiogenic platelets and bone marrow-derived cells. Cancer Discov.

[47] Oskarsson T, Acharyya S, Zhang XHF (2011). Breast cancer cells produce tenascin C as a metastatic niche component to colonize the lungs. Nat Med.

[48] Liu S, Jiang M, Zhao Q (2014). Vascular endothelial growth factor plays a critical role in the formation of the pre-metastatic niche via prostaglandin E2. Oncol Rep.

[49] McAllister SS, Weinberg RA (2014). The tumour-induced systemic environment as a critical regulator of cancer progression and metastasis. Nat Cell Biol.

[50] Ansell SM, Vonderheide RH (2013). Cellular composition of the tumor microenvironment. Am Soc Clin Oncol Educ Book Meeting.

[51] Kareva I (2011). What can ecology teach us about cancer?. Transl Oncol.

[52] Parolini I, Federici C, Raggi C (2009). Microenvironmental pH is a key factor for exosome traffic in tumor cells. J Biol Chem.

[53] Bernhard EJ (2011). Interventions that induce modifications in the tumor microenvironment. Cancer Radiother.

[54] Barcellos-Hoff MH, Lyden D, Wang TC (2013). The evolution of the cancer niche during multistage carcinogenesis. Nat Rev Cancer.

[55] Bussard KM, Smith GH (2012). Human breast cancer cells are redirected to mammary epithelial cells upon interaction with the regenerating mammary gland microenvironment in-vivo. PloS one.

[56] Rosenfield SM, Smith GH (2013). Redirection of human cancer cells upon the Interaction with the regenerating mouse mammary gland microenvironment. Cells.

[57] Dezorella N, Pevsner-Fischer M, Deutsch V (2009). Mesenchymal stromal cells revert multiple myeloma cells to less differentiated phenotype by the combined activities of adhesive interactions and interleukin-6. Exp Cell Res.

[58] Grivennikov SI, Greten FR, Karin M (2010). Immunity, inflammation, and cancer. Cell.

[59] Federico A, Morgillo F, Tuccillo C (2007). Chronic inflammation and oxidative stress in human carcinogenesis. Int J Cancer.

[60] Solinas G, Marchesi F, Garlanda C (2010). Inflammation-mediated promotion of invasion and metastasis. Cancer Metast Rev.

[61] Hanahan D, Weinberg RA (2011). Hallmarks of cancer: the next generation. Cell.

[62] Coussens LM, Werb Z (2010). Inflammation and cancer. Nature.

[63] Cooks T, Harris CC, Oren M (2014). Caught in the cross fire: p53 in inflammation. Carcinogenesis.

[64] Yang L, Karin M (2014). Roles of tumor suppressors in regulating tumor-associated inflammation. Cell Death Differ.

[65] Schwitalla S, Ziegler PK, Horst D (2013). Loss of p53 in enterocytes generates an inflammatory microenvironment enabling invasion and lymph node metastasis of carcinogen-induced colorectal tumors. Cancer Cell.

[66] Staib F, Robles AI, Varticovski L (2005). The p53 tumor suppressor network is a key responder to microenvironmental components of chronic inflammatory stress. Cancer Res.

[67] Hussain SP, Amstad P, Raja K (2000). Increased p53 mutation load in noncancerous colon tissue from ulcerative colitis: a cancer-prone chronic inflammatory disease. Cancer Res.

[68] Cooks T, Pateras IS, Tarcic O (2013). Mutant p53 prolongs NF-κB activation and promotes chronic inflammation and inflammation-associated colorectal cancer. Cancer Cell.

[69] Yamanishi Y, Boyle DL, Pinkoski MJ (2002). Regulation of joint destruction and inflammation by p53 in collagen-induced arthritis. Am J Pathol.

[70] Macedo GS, Lisbôa da Motta L, Giacomazzi J (2012). Increased oxidative damage in carriers of the germline TP53 p.R337H mutation. PLoS ONE.

[71] West XZ, Malinin NL, Merkulova AA (2010). Oxidative stress induces angiogenesis by activating TLR2 with novel endogenous ligands. Nature.

[72] Kim YW, West XZ, Byzova TV (2013). Inflammation and oxidative stress in angiogenesis and vascular disease. J Mol Med.

[73] Dameron KM, Volpert OV, Tainsky MA (1994). Control of angiogenesis in fibroblasts by p53 regulation of thrombospondin-1. Science.

[74] Su F, Pascal LE, Xiao W (2010). Tumor suppressor U19/EAF2 regulates thrombospondin-1 expression via p53. Oncogene.

[75] Ioachim E, Damala K, Tsanou E (2012). Thrombospondin-1 expression in breast cancer: prognostic significance and association with p53 alterations, tumour angiogenesis and extracellular matrix components. Histol Histopathol.

[76] Grossfeld GD, Ginsberg DA, Stein JP (1997). Thrombospondin-1 expression in bladder cancer: association with p53 alterations, tumor angiogenesis, and tumor progression. J Natl Cancer Inst.

[77] Hermeking H (2012). MicroRNAs in the p53 network: micromanagement of tumour suppression. Nat Rev Cancer.

[78] Yamakuchi M, Lotterman CD, Bao C (2010). P53-induced microRNA-107 inhibits HIF-1 and tumor angiogenesis. Proc Natl Acad Sci USA.

[79] De La Peña FA, Kanasaki K, Kanasaki M (2011). Loss of p53 and acquisition of angiogenic microRNA profile are insufficient to facilitate progression of bladder urothelial carcinoma in situ to invasive carcinoma. J Biol Chem.

[80] Volpert O, Dameron KM, Bouck N (1997). Sequential development of an angiogenic phenotype by human fibroblasts progressing to tumorigenicity. Oncogene.

[81] Fontemaggi G, Dell’Orso S, Trisciuoglio D (2009). The execution of the transcriptional axis mutant p53, E2F1 and ID4 promotes tumor neo-angiogenesis. Nat Struct Mol Biol.

[82] Xu J, Qian J, Hu Y (2014). Heterogeneity of Li-Fraumeni syndrome links to unequal gain-of-function effects of p53 mutations. Sci Rep.

[83] Huang Y, Yu P, Li W (2014). P53 regulates mesenchymal stem cell-mediated tumor suppression in a tumor microenvironment through immune modulation. Oncogene.

[84] Shatz M, Menendez D, Resnick MA (2012). The human TLR innate immune gene family is differentially influenced by DNA stress and p53 status in cancer cells. Cancer Res.

[85] Hoebe K, Janssen E, Beutler B (2004). The interface between innate and adaptive immunity. Nat Immunol.

[86] Huang B, Zhao J, Li H (2005). Toll-like receptors on tumor cells facilitate evasion of immune surveillance. Cancer Res.

[87] Rakoff-Nahoum S, Medzhitov R (2009). Toll-like receptors and cancer. Nat Rev Cancer.

[88] Basith S, Manavalan B, Yoo TH (2012). Roles of toll-like receptors in cancer: a double-edged sword for defense and offense. Arch Pharmacal Res.

[89] Sato Y, Goto Y, Narita N (2009). Cancer cells expressing toll-like receptors and the tumor microenvironment. Cancer Microenviron.

[90] Menendez D, Shatz M, Azzam K (2011). The toll-like receptor gene family is integrated into human DNA damage and p53 networks. PLoS genetics.

[91] Yao S, Zhu Y, Chen L (2013). Advances in targeting cell surface signalling molecules for immune modulation. Nat Rev Drug Discov.

[92] Cortez MA, Valdecanas D, Wang X (2015). p53 regulation of PDL1 is mediated through miR-34a. Proc AACR.

[93] Ferreira LMR, Hebrant A, Dumont JE (2012). Metabolic reprogramming of the tumor. Oncogene.

[94] Olovnikov IA, Kravchenko JE, Chumakov PM (2009). Homeostatic functions of the p53 tumor suppressor: regulation of energy metabolism and antioxidant defense. Semin Cancer Biol.

[95] Bonuccelli G, Whitaker-Menezes D, Castello-Cros R (2010). The reverse Warburg effect: glycolysis inhibitors prevent the tumor promoting effects of caveolin-1 deficient cancer associated fibroblasts. Cell Cycle.

[96] Fiaschi T, Marini A, Giannoni E (2012). Reciprocal metabolic reprogramming through lactate shuttle coordinately influences tumor-stroma interplay. Cancer Res.

[97] Martinez-Outschoorn UE, Pavlides S, Howell A (2011). Stromal-epithelial metabolic coupling in cancer: integrating autophagy and metabolism in the tumor microenvironment. Int J Biochem Cell Biol.

[98] Witkiewicz AK, Whitaker-Menezes D, Dasgupta A (2012). Using the ‘reverse Warburg effect’ to identify high-risk breast cancer patients: stromal MCT4 predicts poor clinical outcome in triple-negative breast cancers. Cell Cycle.

[99] Pértega-Gomes N, Vizcaíno JR, Attig J (2014). A lactate shuttle system between tumour and stromal cells is associated with poor prognosis in prostate cancer. BMC Cancer.

[100] Sotgia F, Martinez-Outschoorn UE, Lisanti MP (2014). The reverse warburg effect in osteosarcoma. Oncotarget.

[101] Pantziarka P (2013). Li Fraumeni syndrome, cancer and senescence: a new hypothesis. Cancer Cell Int.

[102] Mercier I, Camacho J, Titchen K (2012). Caveolin-1 and accelerated host aging in the breast tumor microenvironment: chemoprevention with rapamycin, an mTOR inhibitor and anti-aging drug. Am J Pathol.

[103] Sherif ZA, Sultan AS (2013). Divergent control of Cav-1 expression in non-cancerous Li-Fraumeni syndrome and human cancer cell lines. Cancer Biol Ther.

[104] Wang X, Docanto MM, Sasano H (2015). Prostaglandin E2 inhibits p53 in human breast adipose stromal cells : a novel mechanism for the regulation of aromatase in obesity and breast cancer. Cancer Res.

[105] Trkova M, Prochazkova K, Krutilkova V (2007). Telomere length in peripheral blood cells of germline TP53 mutation carriers is shorter than that of normal individuals of corresponding age. Cancer.

[106] Kruk PA, Bohr VA (1999). Telomeric length in individuals and cell lines with altered p53 status. Radiat Oncol Investig.

[107] Pinto C, Veiga I, Pinheiro M (2009). TP53 germline mutations in Portugal and genetic modifiers of age at cancer onset. Fam Cancer.

[108] Tabori U, Nanda S, Druker H (2007). Younger age of cancer initiation is associated with shorter telomere length in Li-Fraumeni syndrome. Cancer Res.

[109] Boyle JM, Mitchell EL, Greaves MJ (1998). Chromosome instability is a predominant trait of fibroblasts from Li-Fraumeni families. Br J Cancer.

[110] Bischoff FZ, Yim SO, Pathak S (1990). Spontaneous abnormalities in normal fibroblasts from patients with Li-Fraumeni cancer syndrome: aneuploidy and immortalization. Cancer Res.

[111] Shay JW, Tomlinson G, Piatyszek MA (1995). Spontaneous in vitro immortalization of breast epithelial cells from a patient with Li-Fraumeni syndrome. Mol Cell Biol.

[112] Tazawa H, Tatemichi M, Sawa T (2007). Oxidative and nitrative stress caused by subcutaneous implantation of a foreign body accelerates sarcoma development in Trp53+/- mice. Carcinogenesis.

[113] Komarova EA, Antoch MP, Novototskaya LR (2012). Rapamycin extends lifespan and delays tumorigenesis in heterozygous p53+/- mice. Aging.

[114] Berrigan D, Perkins SN, Haines DC (2002). Adult-onset calorie restriction and fasting delay spontaneous tumorigenesis in p53-deficient mice. Carcinogenesis.

[115] Eikawa S, Nishida M, Mizukami S (2015). Immune-mediated antitumor effect by type 2 diabetes drug, metformin. Proc Natl Acad Sci USA.

[116] Pantziarka P, Bouche G, Meheus L (2014). The repurposing drugs in oncology (ReDO) project. Ecancermedicalscience.

